# A Review on Polymers for Biomedical Applications on Hard and Soft Tissues and Prosthetic Limbs

**DOI:** 10.3390/polym15194034

**Published:** 2023-10-09

**Authors:** Heitor Luiz Ornaghi, Francisco Maciel Monticeli, Lucas Dall Agnol

**Affiliations:** 1 Mantova Indústria de Tubos Plásticos Ltd.a., R. Isidoro Fadanelli, 194-Centenário, Caxias do Sul 95045-137, RS, Brazil; 2 Department of Aerospace Structures and Materials, Faculty of Aerospace Engineering, Delft University of Technology, 2628 CD Delft, The Netherlands; f.m.monticeli@tudelft.nl; 3 Postgraduate Program in Materials Science and Engineering (PGMAT), University of Caxias do Sul, Caxias do Sul 95070-560, RS, Brazil; lucasdall1989@hotmail.com

**Keywords:** polymers, biomedical, bone fracture repair, soft and hard tissue, dental applications, prosthetic limbs

## Abstract

In the past decades, there has been a significant increase in the use of polymers for biomedical applications. The global medical polymer market size was valued at USD 19.92 billion in 2022 and is expected to grow at a CAGR of 8.0% from 2023 to 2030 despite some limitations, such as cost (financial limitation), strength compared to metal plates for bone fracture, design optimization and incorporation of reinforcement. Recently, this increase has been more pronounced due to important advances in synthesis and modification techniques for the design of novel biomaterials and their behavior in vitro and in vivo. Also, modern medicine allows the use of less invasive surgeries and faster surgical sutures. Besides their use in the human body, polymer biomedical materials must have desired physical, chemical, biological, biomechanical, and degradation properties. This review summarizes the use of polymers for biomedical applications, mainly focusing on hard and soft tissues, prosthetic limbs, dental applications, and bone fracture repair. The main properties, gaps, and trends are discussed.

## 1. Introduction

Biomaterials (BM) can be defined as a synthetic or natural material suitable for use in direct interaction with the human body as in the construction of artificial organs, prostheses, or replacement bone or tissue. BMs are a multi-disciplinary research area that involve both materials science and medicine fields, combining the mechanical performance of the material with the human body compatibility. BMs can be synthetic or natural and can replace some or all parts of the human body [1]. Advances in technology allow for the obtaining of new tissues or organs of high quality that are accepted by the human body (they must have specific properties to be long-lasting). BMs are applied, but not limited to, to promote healing of human tissues (scaffold cells and bioactive molecules), drug delivery systems (creating new routes via pulmonary, transdermal, ocular, and nasal routes), medical implants (heart valves, ligaments, dental implants), nanoparticle biosensors (based on gold nanoparticles, semiconductor quantum dots, nanodiamonds), and molecular probes (digoxigenin, porphyrin, cyanine).

**Figure 1:**
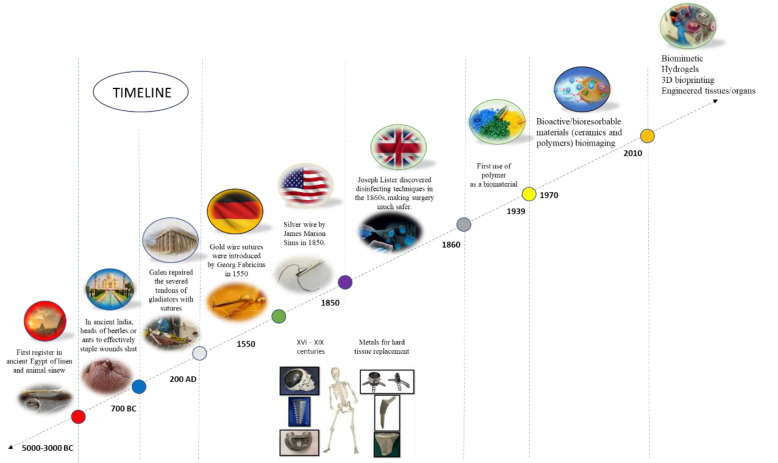
Schematic representation of the timeline indicating some of the main data and the respective discoveries.

The first register of biomedical materials [2] dates to between 5000 and 3000 BC, when Egyptians employed linen and animal sinew to close wounds. Also, in ancient India, the heads of beetles or ants were used for the same purpose. The physician cut the insects’ bodies off, leaving the jaws in linen strips coated with resin or with an adhesive mixture of honey and flour, which appeared in ancient Egyptian medicine as forerunners of our modern-day skin closure tapes. Cornelius Celsus described small metal clips for closure. Aelius Galenus, a physician, surgeon, and philosopher of the ancient Roman Greeks, repaired the damaged tendons of gladiators with sutures. Gold and silver sutures were introduced in 1550 and 1850, respectively. In the 1860s, Joseph Lister discovered disinfecting techniques, which helped decrease mortality. Catgut sutures treated with chromium salts were used as sutures. Recent developments showed bioactive and bioresorbable materials for bioimaging and cancer therapy, biomimetic 3D bioprinting, and engineered tissues and organs. With the increasing demand for new materials and the recent advances in technology and science, even more materials are being created with better performance than conventional ones [1,3]. Figure 1 shows a schematic representation of the timeline of the BMs with some important data and the respective materials. More than ever, the increasing demand for polymer in biomedical applications requires new insights or interpretations as well as the main novelties and trends in the area. By compilating this information, it is expected that the most important information on this topic will be contained in a single document which can be a first guide for researchers and scientists.

Data from 2021 have shown that the global biomaterials market size is 135.4 billion dollars, with orthopedic, dental, and cardiovascular being more than 50% of all BMs. Neurology, plastic surgery, ophthalmology, wound healing, tissue engineering and others compose 50% of the materials. An expected growth of 17% UK CAGR (compound annual growth rate) is expected from 2022 to 2030 formed by polymers, metallic, natural, and ceramics materials. According to Medical Polymer Market Size, Share and Trends Analysis Report [4], the global medical polymer market size was valued at USD 19.92 billion in 2022 and is expected to grow at a CAGR of 8.0% from 2023 to 2030. This trend is mainly motivated by the huge number of medical-grade polymers that are more versatile than most of the conventional materials used for the same application. The global medical polymer market considers materials such as medical components, medical device packaging, mobility aids, sterilization and infection prevention, other implants, biopharma devices, cleanroom supplies, wound care, orthopedic soft goods, and others. It is noteworthy to mention that most of the polymers are fibers and resins while a lower amount is attributed to biodegradable polymers. Table 1 shows the mechanical properties of some typical materials used in the human body for different functions. It is notorious that for low-modulus materials, hydrogels are more recommended while for high-modulus materials inorganic materials are indicated.

Recent progress in biomedical applications includes several interesting studies, including some commercially available products. One example is RESOMER@ commercially available by Evonik Rohm GmbH which includes poly (glycolic acid) (PGA), poly (lactic acid) (PLA) and their copolymers. For the 3D printing of tissue engineering scaffolds, a vast variety of polymers are available on the website as printing filaments (most of them) [5].

**Table 1:** Mechanical properties of hard tissue, soft tissue, typical metallic alloys, and polymers [3,6,7,8,9].

Classification	Material Type	Modulus (GPa)	Tensile Strength (MPa)
Hard Tissue	Cortical bone (longitudinal direction)	10.0–30.0	100.0–150.0
	Cortical bone (transverse direction)	10.0–30.0	1–50.0
	Cancellous bone	0.1–5.0	5.0–20.0
	Enamel	60.0–90.0	8.0–10.0
	Dentine	10.0–20.0	30.0–40.0
Soft Tissue	Articular cartilage	0.5–10.5	0.5–27.0
	Fibrocartilage	1.0–10.0	2.0–12.0
	Ligament	0.1–1.0	20.0–60.0
	Tendon	0.4–1.5	46.0–100.0
	Skin	1 × 10^−4^–1.0	10.0–20.0
	Arterial tissue (longitudinal direction)	–	0.1–0.5
	Arterial tissue (transverse direction)	–	1.0–5.0
	Intraocular lens	5 × 10^−3^–3.0	2.0–40.0
Metal alloys	Stainless steel	1 × 10^−2^–10.0	500.0–2000.0
	Co-Cr alloy	0.4–0.6	900.0–1100.0
	Ti-alloy	2.0–3.0	900.0–1100.0
	Amalgam	30	50.0–300.0
Ceramic	Alumina	300–400	300.0–500.0
	Zirconia	200–300	800.0–1200.0
	Bioglass	30–50	40.0–200.0
	Hydroxyapatite	90–100	50.0–130.0
Polymer	Polyethylene (PE)	0.1–1.5	10.0–50.0
	Polyurethane (PU)	1 × 10^−2^–10.0	20.0–70.0
	Polytetrafluorethylene (PTFE)	0.4–0.6	20.0–40.0
	Polyacetal (PA)	2.0–3.0	50.0–90.0
	Polymethylmethacrylate (PMMA)	2.0–3.0	50.0–100.0
	Polyethylene terephthalate (PET)	2.0–3.0	50.0–100.0
	Polyetheretherketone (PEEK)	3.0–8.0	90.0–140.0
	Silicone rubber (SR)	8 × 10^−3^–0.5	5.0–20.0
	Polysulfone (PS)	2.0–3.0	60.0–90.0
	Polycaprolactone (PCL)	0.1–1.0	10.0–40.0
	Poly(lactic acid) (PLA)	2.0–5.0	40.0–80.0
	Poly(glycolic acid) (PGA)	3.0–6.0	50.0–100.0
	Poly(lactic-co-glycolic acid) (PLGA)	2.0–8.0	30.0–80.0
	Polydioxanone (PDO)	1.0–5.0	40.0–70.0
	Polypropylene (PP)	1.0–2.0	20.0
	Polycarbonate (PC)	2.0–3.0	60.0–80.0
	Polysaccharides (e.g., chitosan)	0.1–1.0	5.0–20.0
	Hydrogels (e.g., alginate, gelatin)	0.01–1.0	0.1–10.0
	Poly(ε-caprolactone-co-lactide) (PCLA)	0.5–5.0	5.0–30.0

The incorporation of nanoparticles such as clay [10] or curcumin-based hydroxyapatite [11], into biomaterials, can give new or activated therapeutic properties [12]. One of the most important applications of nanoparticles (NPs) is for clinical applications, overcoming limitations of free therapeutics and navigating biological barriers (systemic, microenvironmental and cellular) that differ from patient populations and diseases. Different classes of NPs are found: polymeric (polymersome, dendrimer, polymer micelle, nanosphere), inorganic (silica, quantum dot, iron oxide, gold), lipid-based (liposome, lipid, emulsion). Each class of NP has its advantages and disadvantages regarding cargo, delivery, and patient response. The best alternative would be a one-size-fits-all-solution hence much effort is put into this issue to enhance therapeutic efficacy [13].

This mini-review shows some of the main applications of BMs used as bone fracture repair, soft and hard tissues, and prosthetic limbs. It shows the main applications and some of the main developments about this topic.

## 2. Hard Tissue Applications

### 2.1. Bone Fracture Repair

**Figure 2:**
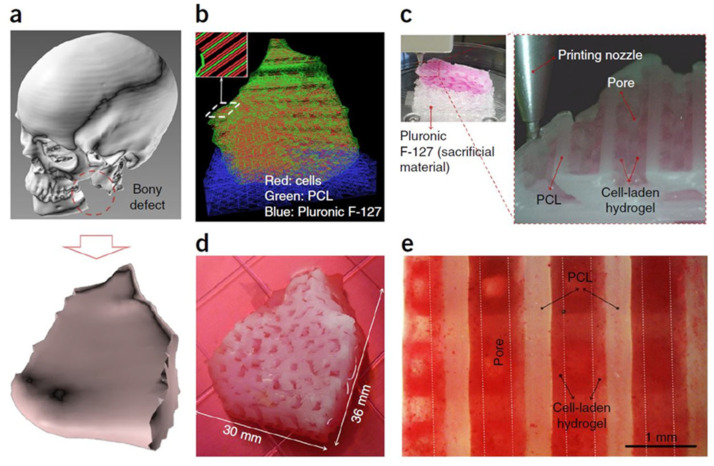
Bone reconstruction of the mandible using polymer material. Reproduced with permission from Ref. [24]. Copyright © 2016, Springer Nature America, Inc. (**a**) 3D CAD model recognized a mandible bony defect from human CT image data. (**b**) Visualized motion program was generated to construct a 3D architecture of the mandible bone defect using CAM software developed by our laboratory. Lines of green, blue and red colors indicate the dispensing paths of PCL, Pluronic F-127 and cell-laden hydrogel, respectively. (**c**) 3D printing process using the integrated organ printing system. The image shows patterning of a layer of the construct. (**d**) Photograph of the 3D printed mandible bone defect construct, which was cultured in osteogenic medium for 28 d. (**e**) Osteogenic differentiation of hAFSCs in the printed construct was confirmed by Alizarin Red S staining, indicating calcium deposition. The same legend of the original study was maintained.

Fractures, both traumatic and pathological, necessitate innovative approaches to promote efficient bone healing and minimize complications [14]. Polymer materials offer versatile solutions for fracture repair, serving as scaffolds to support tissue regeneration [15], carriers for therapeutic agents [16], and vehicles for targeted drug delivery [17]. This section provides an overview of the polymers used as structures to address bone fractures, encompassing fabrication techniques, structural design considerations, and functional modifications. Polymer-based scaffolds provide a three-dimensional framework that mimics the natural extracellular matrix of bone tissue, facilitating cell attachment, proliferation, and differentiation [18]. These scaffolds, typically composed of biocompatible polymers, provide a three-dimensional structure that mimics the extracellular matrix of natural bone tissue [19,20]. One of the key advantages of polymer-based scaffolds is their tunable mechanical properties, allowing them to match the stiffness and strength of native bone, as shown in Table 1 [3,6]. This feature is critical for providing structural support to the injured site and facilitating load-bearing during healing [21]. Besides the advantages of creating the appropriate mechanical behavior using polymer composites, polymers are generally lighter than conventional metals, making them a favorable choice for implants used in bone fracture repair [22]. The lightweight nature of polymers reduces the overall weight burden on the body, which can be especially beneficial in load-bearing applications. This characteristic helps minimize stress on the surrounding tissues and facilitates patient comfort and mobility during the repair process [23]. Figure 2 illustrates an application example conducted by Kang et al. [24] where they reconstructed a mandible bone using a composite hydrogel composed of polycaprolactone (PCL), tricalcium phosphate (TCP), and human amniotic fluid stem cells (hAFSCs).

The hydrogel was 3D printed in a type I pattern with temporary support using Pluronic F127 (powder, BioReagent, suitable for cell culture). After 28 days of osteogenic differentiation, Alizarin Red S staining confirmed calcium deposition in the hAFSC-laden hydrogel, indicating successful bone formation. Hyaluronic acid-based nanocomposites were found to facilitate cell organization, proliferation, and nutrient transport in developing tissues. They also provide ear cartilage and skeletal muscle reconstruction by applying a similar methodology.

Metals typically have a higher coefficient of thermal expansion than polymers. When a metallic implant is subjected to temperature changes, such as during hot or cold environments, it can expand or contract significantly [25,26]. This difference in thermal expansion may cause stress on the surrounding bone tissue and compromise the healing process. In contrast, polymers generally have lower coefficients of thermal expansion, which can help minimize the adverse effects of temperature variations on the implant-bone interface [27].

#### 2.1.1. Porous Materials

The use of porous polymer materials for the healing of fractured bones holds significant importance and offers several advantages in the field of bone repair. Porous structures in polymer materials provide interconnected voids and channels that facilitate the infiltration and proliferation of cells, including osteoblasts and mesenchymal stem cells [28,29]. These cells are crucial for bone regeneration and can populate the pores of the polymer scaffold, promoting the formation of new bone tissue. The porous architecture of the polymer scaffold supports the diffusion of nutrients and oxygen, providing a conducive environment for cell survival and growth. Porous polymer materials can also be designed to mimic the architecture of natural bone, including the trabecular or cancellous bone structure. The interconnected pores and open spaces in the scaffold closely resemble the porosity and interconnectivity found in cancellous bone, enabling better integration between the scaffold and surrounding bone tissue. This biomimetic approach enhances the mechanical stability of the repair site and facilitates the transfer of load-bearing forces during the healing process [23,30,31]. In addition, vascularization, the formation of new blood vessels, is critical for successful bone regeneration. Porous polymer scaffolds with interconnected pores allow for the ingrowth of blood vessels, supporting the establishment of a vascular network within the healing site. Adequate vascularization supplies oxygen, nutrients, and growth factors to the regenerating tissue, promoting faster and more efficient bone healing [26].

**Figure 3:**
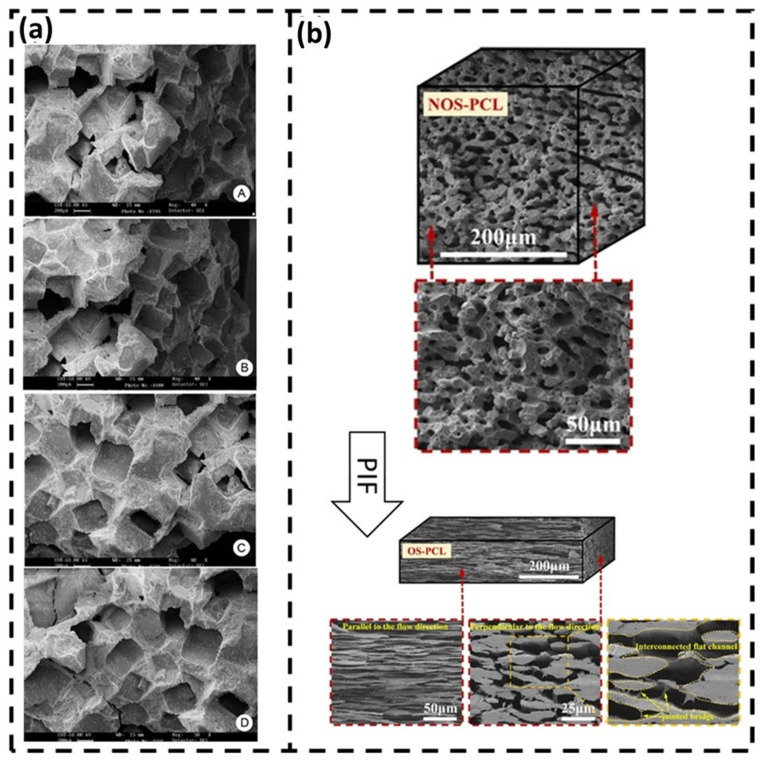
(**a**) porous multicomponent bio-nanocomposite acting as a biocompatible polymer substrate to support bone (Reproduced with permission from Ref. [32]. Copyright © 2021 Elsevier Ltd. and Techna Group S.r.l.) and (**b**) micrographs of NOS-PCL and OS-PCL scaffolds (Reproduced with permission from Ref. [33]. Copyright © 2023 Springer Nature).

The porosity of the polymer scaffold can be precisely controlled (Figure 3), enabling the customization of the mechanical properties and degradation kinetics to match the specific requirements of the bone healing process.

Adjusting the pore size, distribution, and overall shape can influence cell infiltration, nutrient diffusion, and mechanical support. This tunability allows for the optimization of scaffold properties based on the type and location of the fracture, as well as the patient’s individual needs [33]. Additionally, porous polymer scaffolds offer the advantage of incorporating bioactive agents, such as growth factors, antibiotics, or anti-inflammatory drugs, within the porous structure. The high surface area and interconnected porosity of the scaffold facilitate the controlled release of these agents, providing localized and sustained delivery to the healing site [34]. This approach promotes osteogenic activity, reduces infection risks, and modulates the inflammatory response, further enhancing the bone healing process. The porous nature of these scaffolds promotes cell infiltration, nutrient diffusion, and vascularization, which are essential for the successful regeneration of bone tissue [33].

#### 2.1.2. Polymer-Based Scaffolds

Polymer-based scaffolds also offer versatility in their design and fabrication. Several techniques enable the creation of scaffolds with controlled architecture, pore size, and interconnectivity, such as electrospinning, 3D printing, and self-assembly [35]. This allows for tailoring the scaffold properties to meet specific requirements, such as promoting cell attachment, proliferation, and differentiation. Furthermore, these structures can be functionalized with bioactive molecules, such as growth factors or peptides, to enhance their osteoinductive and osteoconductive properties [36]. This provides an additional advantage in promoting cellular responses and accelerating the formation of new bone at the fracture site [37].

However, certain limitations are associated with polymer-based scaffolds or structures for bone repair. One significant limitation is the degradation rate of the polymer materials. Ideally, the scaffold should degrade at a rate that matches the rate of new bone formation [38]. If the degradation is too slow, it may hinder the regeneration process, while rapid degradation can compromise the scaffold’s mechanical integrity before sufficient new tissue is formed. Achieving the optimal balance between scaffold degradation and new bone formation remains challenging [34,38].

Another limitation lies in the lack of innate biological signals within the polymer scaffolds. While the architecture and surface modifications of the scaffolds can enhance cellular responses, they may only partially replicate the complex biochemical cues present in natural bone tissue [39]. Incorporating signaling molecules and growth factors within the scaffold or combining the scaffold with other BMs, such as ceramics or bioactive coatings, can address this limitation and provide a more biomimetic environment for bone repair [40].

**Figure 4:**
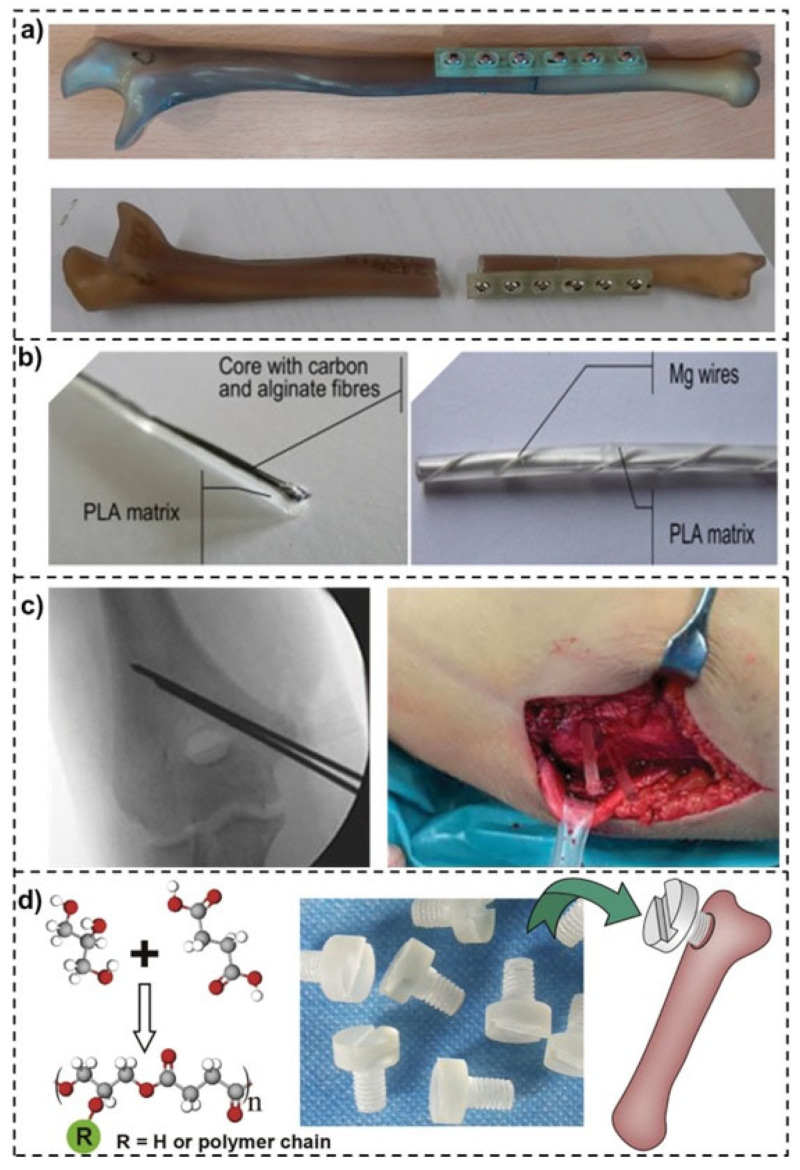
(**a**) Polymer-based composite plated broken bones [41], (**b**) Polymer based nails: Polylactic acid (PLA) + Long carbon fibers (CF) + Long calcium alginate fibers (Alg) + Gentamicin sulfate (GS (S)) [39], (**c**) Transient stabilization with Kirschner wires (K-wires) replaced with biodegradable pins [42], and (**d**) Poly(Glycerol Sebacate) (PGS) synthesized from glycerol and succinic acid applied as screw in bone repair [50]. (with kindly permission from Elsevier and National Library of medicine). Subfigures (**a**) and (**c**) were re-used under the Creative Common CC-BY license.

Several structures are used to repair fractured bones, exhibiting a wide range of applications. One example is the bone plates that are usually made from metallic materials and could be replaced by polymer-based, offering significant advantages in bone repair (Figure 4a). One key advantage is their lightweight nature (i.e., 70% lighter and 25% shorter), which reduces the overall implant load and minimizes stress shielding effects on the surrounding bone [41]. Additionally, polymers can closely match the mechanical properties of bone, such as elasticity, leading to reduced stress concentrations at the fracture site [22]. This property promotes better load transfer and can improve the healing process. Poly-L-lactic acid (PLLA), polycaprolactone (PCL), and polyetheretherketone (PEEK) are commonly used polymers in bone plates [42]. However, these materials do have limitations. They exhibit lower strength compared to metal plates, making them more prone to fatigue fracture [42]. Ensuring the long-term stability and durability of polymer-based bone plates requires addressing these limitations through careful material selection, design optimization, and incorporating reinforcements such as fibers or additives [41].

Another application for polymer-based structure is the intramedullary nail, which proposes several advantages in fractured bone repair (Figure 4b). They provide internal fixation and stability while exhibiting reduced stiffness compared to traditional metal nails [43]. This reduced stiffness helps to mitigate stress shielding and allows for more physiological loading on the healing bone. Additionally, polymers like polyetheretherketone (PEEK), polylactide (PLA) and their composites have demonstrated excellent biocompatibility and can promote bone healing [43,44]. However, challenges remain in optimizing the mechanical properties of polymer-based intramedullary nails. Balancing flexibility and strength is crucial to ensure adequate stabilization and prevent implant failure. Further research is needed to refine the mechanical characteristics and degradation profiles to ensure long-term performance and enhance their potential in fractured bone repair [45].

#### 2.1.3. Polymer-Based Kirschner-Wires (K-Wires)

Polymer-based K-wires offer advantages in terms of reduced invasiveness, minimized soft tissue damage, and decreased risk of stress risers [46]. These implants are commonly used for fracture fixation and stability. Biodegradable polymers, such as polylactide (PLA) and polyglycolide (PGA), have gained attention in this field due to their gradual degradation over time, eliminating the need for implant removal, as shown in Figure 4 [46,47]. This property reduces the risk of complications associated with permanent implants. Nevertheless, the mechanical strength of polymer-based K-wires needs further optimization. Balancing the degradation kinetics with the required mechanical properties is crucial for ensuring adequate stability during the healing process [48]. Ongoing research aims to address these challenges and harness the full potential of polymer-based K-wires in fractured bone repair applications [49].

#### 2.1.4. Polymer-Based Screws

Polymer-based screws have also gained significant attention in fractured bone repair due to their unique advantages (Figure 4d). One key advantage is their reduced risk of corrosion and implant-related infections compared to traditional metal screws [50]. This is particularly beneficial for long-term implantation, as it minimizes the chances of implant failure and the need for revision surgeries. Additionally, polymer screws offer improved biocompatibility, reducing the risk of adverse reactions or inflammatory responses in the surrounding tissue [4]. Another advantage of polymer screws is their ability to provide adequate stability while being gentler on the bone [50]. On the other hand, polymer-based screws exhibit lower stiffness compared to metal screws, allowing for more physiological loading and stress distribution at the fracture site. This can help to promote proper bone healing and prevent stress shielding, which occurs when the implant bears too much of the load, resulting in reduced bone density and weakened healing [51].

There are some limitations associated with polymer screws that need to be addressed. One of the main challenges is optimizing the mechanical strength of the polymer material to ensure sufficient stability and fixation [52], as aforementioned. The mechanical properties of polymers can be tailored through various methods, such as incorporating reinforcements or optimizing the material composition. Balancing the need for strength with the desired degradation profile of the polymer is crucial to ensure long-term performance [53].

#### 2.1.5. Other Applications

**Figure 5:**
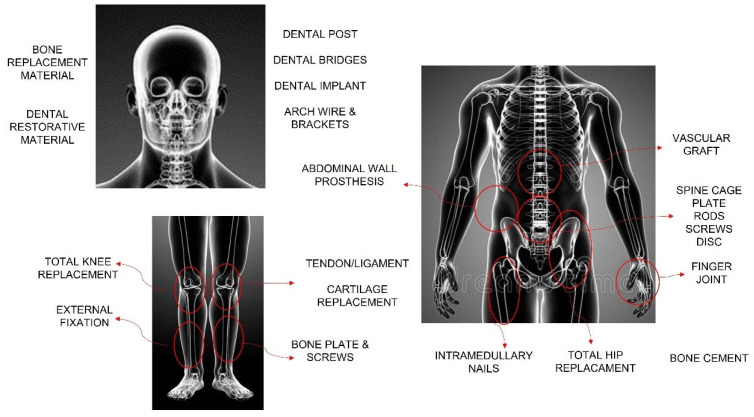
Schematic representation of the application of polymers in the human body. Adapted from Ref. [3]. Copyright © 2001 Elsevier Science Ltd.

Other applications such as bone plates, intramedullary nails, spine instrumentation, joint replacements, total hip replacements, total knee replacement, and other joint replacements, i.e., bone cements, among others, are schematically represented in Figure 5. Figure 5 represents some of the applications of the biopolymers in the human body. It is important to mention that the BMs are from hydrogels, and elastomers to inorganic materials, being used as hard (bone) or soft (muscle) tissue, bone repair fracture, and prosthetic limbs, among others. More details can be found in Ramakrishna et al. [3].

### 2.2. Dental Applications

**Figure 6:**
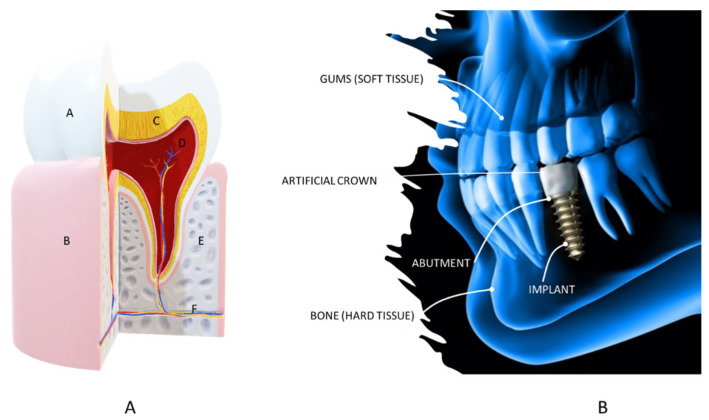
(**A**). Anatomy of the tooth where A is the enamel, B is the gums, C is the dentin, D is the pulp, E is the alveolar bone, and F are the nerves and blood vessels (picture from the own authors based on [54] and (**B**). Lateral view of the dental implant (figure based on [55]).

The tooth is composed of four main dental tissues. Enamel, dentin, and cementum are hard tissues while the pulp is a soft tissue. Also, the tooth can be divided into two main portions: crown and root. Figure 6 represents the anatomy of the tooth with the main components. It can be noted that the tooth is composed of both hard and soft tissues.

In a study by Healthy Roots Dentistry, Tulsa, USA [56] it was concluded that each tooth in the human mouth is related by a meridian to an organ of the body and that the organs can be affected by dental health. For example, gum disease can be directly related to the heart because when the gum is infected, it leaks bacteria and toxins into the bloodstream. Hence, it is very important to prevent and care for the tooth to avoid some diseases. In a more severe case, the replacement of the tooth by BMs. In a mini-review of dental implant biomaterials by Semisch-Dieter et al. [49] different types of materials were described: titanium, alternative alloys, zirconia, bioactive glass (BAG), and polymers. Regarding BAG, some studies can be found in the literature; Raszewski et al. [57] studied the preparation and characterization of acrylic resin using 10% bioactive glasses. The authors claimed that the acrylic material modified with bioactive glass met the regularization of ISO 20795-1:2013 in terms of flexural strength and sorption. Also, the bioactive glass releases calcium and silicon phosphor ions after a period of 42 days. Tiskaya et al. [58] published a critical review about the use of BAG in dental composites using online databases (Science Direct, PubMed and Google Scholar) to collect data from 1962 to 2020. The major benefit in dentistry includes their capacity to form apatite (at the cost of releasing Ca^2+^, PO_4_^3−^ and F^−^ ions and raising pH), which potentially fills any marginal gaps produced due to polymerization shrinkage. Other alternative materials such as graphene, magnesium composite and ceramic composites were also described. Specifically, polymers containing fibers (FRC–fiber reinforced composites) have many benefits over conventional materials. Besides their excellent mechanical performance, the biocompatibility and osseointegration shown to be comparable to pure titanium. Another advantage lies in the implant which can be moldable in situ. The stress-transferring mechanism occurs in the fiber direction from the outer layer to the inner substrate. Maybe the most promising polymer is polyetheetherketone (PEEK), which according to the authors, when reinforced by fiber, can replicate the biomechanics of human cortical bone, potentially decreasing bone loss and improving osseointegration. The lower fracture resistance compared to pure titanium is compensated for by the decreased stress shielding. One of the future trends is the use of collagen hydrogels, which can favor biocompatibility and decrease cytotoxicity. These hydrogels can simulate a 3D living microenvironment and fill any shape damage ability and have been extensively applied in tissue engineering [59,60].

Different materials are used for dental implants, the most common are Ti and Ti-6Al-54V, ceramic materials (alumina, zirconia) [60]. However, poly-ether-ether-ketone (PEEK) has been successfully employed in dental applications due to some advantages such as being colorless and endowed with an elastic modulus close to that of the bone, as presented in Table 2.

**Figure 7:**
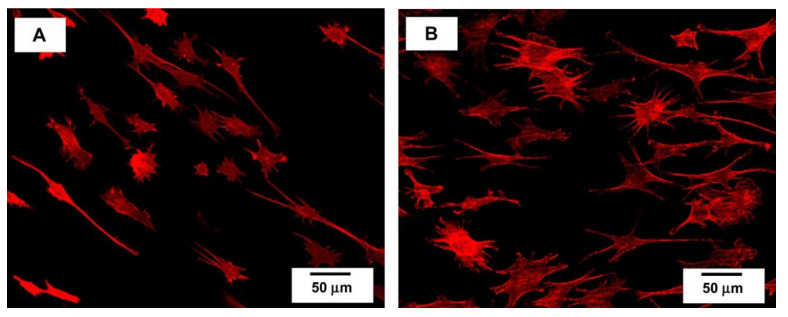
Confocal laser scanning microscopy (CLSM) images of the MC3T3-E1 cell cultured on (**A**) as-machined and (**B**) Ti-coated PEEK for 3 h. Reproduced with permission from Ref. [62]. Copyright © 2009 Elsevier Ltd.a.

Figure 7 shows PEEK coated with Ti using the e-beam deposition technique. It demonstrates good cell attachment, with the red color representing the actin in the cells, indicating good biocompatibility. However, the cells appeared to grow and spread more actively on the Ti-coated PEEK substrate (Figure 7B) than on the pure PEEK substrate (Figure 7A) [62].

The use of polymers for dental applications has to englobe a brilliant surface combined with ideal mechanical and organic properties [63]. This area can be divided into different uses: (i) prosthodontics (concerned with the impact of tooth or tissue damage and partial or complete loss of teeth on oral function in its broadest sense) [64], (ii) operative dentistry (diagnosis, prevention, treatment, and prognosis of diseases or trauma of the dentition) [65], (iii) orthodontics (alignment of the bite and straightening of the teeth) [66], (iv) endodontics (concerning dental pulp and tissues surrounding the roots of a tooth) [67], (v) equipment (mixing bowls and spatulas, mouth guards and defensive eyewear) [61]. Polymethyl methacrylate (PMMA) is the most used polymer for denture base material. For soft lining materials, two sorts of materials can be used: delicate acrylics and silicone rubbers. In the case of dental composites, the materials are engineered polymers with particulate ceramic fillers UV-cured. Polyethers and polysulfides can be used as impression materials while polyetheretherketone (PEEK) and polyaryletherketone (PAEK) are widely used for interminable prosthetic modifying endeavors. Another alternative is the bioactive PEEK with ceramic filler which has optimal cleaning properties and low plaque affinity.

Most of the materials used in the implantology field are metals or alloys. Implantology involves the osseointegration of titanium or other metal inserts with surface alterations using fiber-reinforced fillers as hydroxyapatite or antimicrobial circuit by method for thermoset polymers [63], in cases involving polymers. Another example is the polymer/carbon-fiber composite. In respect to polymer-based bone grafts in dentistry, the material can have degradable or nondegradable characteristics, such as the open porosity polylactic acid polymer [68].

## 3. Soft Tissue Applications

**Figure 8:**
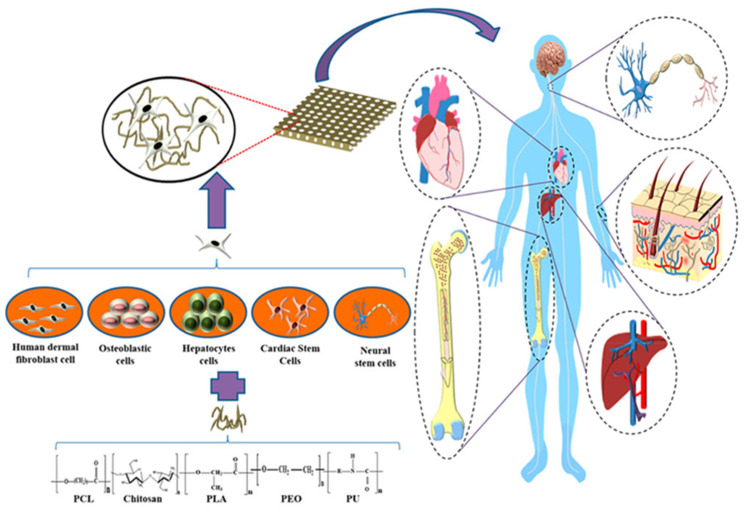
Schematic representation of some polymers combined with different cells to build a scaffold for different applications in the human body. Reproduced with permission from [59].

Soft tissues connect and support other tissues and surround the organs in the body. They include skin, muscle, tendons, fat, nerves, blood vessels, ligaments, and other fibrous tissues. Soft tissues are easily damaged compared to hard tissues and hence new strategies have been developed aiming to fast repair and regenerate the damaged tissues. Also, it is noteworthy to mention that several cell types are also presented due to the diversity of the soft tissues found in the human body. Adult cells (keratinocytes, fibroblasts, adipocytes) or adult stem cells (mesenchymal stem cells) are usually loaded in biocompatible scaffolds [69]. In this sense, BMs are an excellent alternative to building scaffolds with appropriate structures and tailored functionalities that can support cell growth and new tissue formation. The combination with other synthetic or inorganic materials is also an alternative. The direct replacement of the damaged soft tissue is still commonly applied in current clinical practices conducted using inert implants or autologous grafts causing adverse effects, such as chronic pain and implant-related complications. In comparison, scaffolds in two-dimensional or three-dimensional forms can be used as templates for tissue regeneration. The cells can bind to the scaffolds and then proliferate and differentiate. In addition, growth factors can be incorporated into the scaffolds to advance tissue regrowth and repair. To meet the clinical needs, the scaffolds for soft tissue repair should have tissue-matching mechanical properties, excellent biocompatibility, and appropriate biodegradability. Both synthetic polymers and natural polymers have been used to fabricate scaffolds. The synthetic polymers include polylactic acid (PLA), polyglycolic acid (PGA), poly (lactic-co-glycolic acid) (PLGA), and poly-ecaprolactone (PCL), while the natural polymers include proteins and polysaccharides. Compared with synthetic polymers, natural polymers such as collagen, fibrin, silk protein, chitosan, and hyaluronic acid generally present better biocompatibility but limited processability. With the rapid development of processing technology in recent years, more natural polymer-based scaffolds have been successfully fabricated and applied in biomedical applications. Table 3 summarizes the status of the natural polymer-based scaffolds that are commercially available or in clinical trials. The main components of these scaffolds are primarily composed of fibrinogen, collagen, silk, and alginate. The materials can be fabricated into functional scaffolds for various applications, including wound repair, hernia repair, cartilage repair, and blood vessel grafting. In some cases, the repairing efficacy can be improved by incorporating bioactive materials such as growth factors and antibacterial agents in the scaffolds. In recent years, silk-based scaffolds have attracted much attention due to their excellent mechanical properties and biocompatibility. The main applications are schematically presented in Figure 8.

**Table 3:** Status of natural polymer-based scaffolds in clinical use/translation [Data obtained from Ref. [69].

Trade Name/Product Name	Materials	Company/Institution	Applications
Chongshu^®^ composite hernia patch	Fibrinogen; poly (lactide-co-epsilon-caprolactone)	Shanghai Pine and Power Technology Co., LTD	Hernia repair
Haiao^®^ oral repair membrane	Collagen	Yantai Zhenghai Biotechnology o. LTD	Peridontal tissue repair
GenossDES^TM^	Cobalt-chromium platform scaffolds containing sirolimus biodegradable polymers	Genoss Company Limited, Suwon, Korea	Coronary stent implantation
BEGO^®^ collagen membrane	Collagen membrane	BEGO Implant Systems	Tissue engineering
Mucograft	Collagen types I and III	Geistlich Pharma AG, Wolhusen, Switzerland	Gingival recession
Collagen Graft and Collagen Membrane	Collagen Membrane, Collagen Graf	Genoss Company Limited, Suwon, Korea	Cleft palate repair
PACCG-GelMA Hydrogels	Poly (N-acryloyl 2-glycine)/methacrylated gelatin hydrogels	Tianjin Key Laboratory of Composite and Functional Materials	Osteochondal regeneration
PEG silk composite hydrogel	Silk	Research Institute of Agriculture and Life Sciences, Seoul National University, Seoul, South Korea	Articular cartilage repair
Elastin-silk fibroin double raschel knitted vascular graft	Silk	Tokyo University of Agriculture and Technology, Fuchu, Japan	Artificial blood vessel
Chondrotissue^®^	PGA, HA	Chondrotissue, BioTissue, AG, Zurich, Switzerland	Cartilage tissue engineering
IC scaffold	PLGA, COL	Tissue Engineering Research Center, AIST Kansai, Amagasaki Site	Cartilage tissue engineering
C2C1H scaffold	PLA, COL, CH	BioMediTech, Institute of Biosciences and Medical Technology, Tampere, Finland	Cartilage tissue engineering
Chitosan-modified PLCL scaffold	PLCL, CH	Tissue Engineering Program, Life Sciences Institute, National University of Singapore, Singapore	Cartilage tissue formation
CSMA/PECA/GO (S2) scaffold	CSMA, MPEG-PCL-AC (PECA), GO	State Key Laboratory of Biotherapy and Cancer Center, West China Hospital, Sichuan University	Cartilage tissue engineering
Hyalofast^®^	Benzyl ester of hyaluronic acid	Anika Therapeutics Inc., Bedford, Massachusetts, United States	Osteochondral Injury
ChondroGide^®^	Type I/III collagen	Geistlich Biomaterials, Wolhusen, Switzerland	Cartilage defects of the knee joint
Cartipatch^®^	Agarose and alginate	Tissue Bank of France, TBF, Lyon, France	Knee cartilage injury
Silk Voice^®^	Silk	Sofregen, United States	Wound healing
NOVOCART^®^ 3D	Type I collagen, chondroitin sulfate	TETEC, Reutlingen, Germany	Isolated retro patellar cartilage defects

Chitosan (CH); collagen (COL); methacrylated chondroitin sulfate (CSMA); hyaluronic acid (HA); polycaprolactone (PCL); polylactic acid (PLA); poly (l-lactide) (PLLA); poly (glycolic acid) (PGA); polylactic-co-glycolic acid (PLGA); extracelular matrix (ECM); poly (l-lactide-co-ε-caprolactone) (PLCL); acryloyl chloride (AC); graphene oxide (GO).

**Figure 9:**
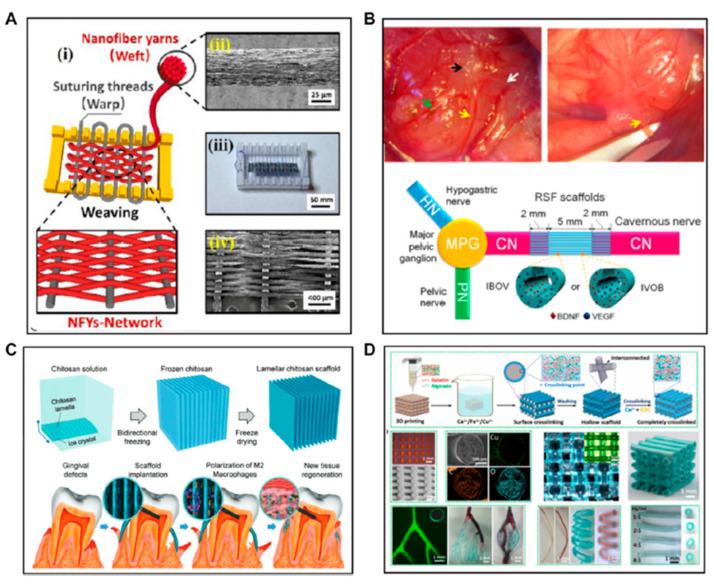
The fabrication of natural polymer-based scaffold via (**A**,**B**) Electropinning, (**C**) Freeze-drying and (**D**) 3D printing. The figure was obtained by kind permission of [70]. The legend and the description were kept the same as in the original study. (**A**) is related to the scaffolds with a core of RSV-VEGF (Respiratory syncytial virus-vascular endothelial growth factor) and a shell of RSF BDNF (regenerated silk fibroin- brain-derived neurotrophic factor) promoting the regeneration of cavernous nerves. (**B**) shows a scaffold within a hydrogel shell composed of aligned electrospun conductive nanofibers which contained polycaprolactone, silk fibroin, and carbon nanotubes. Cardiomyocytes were aligned along the nanofibers on each layer of the 3D nanofibrous scaffold in the stable hydrogel environment. (**C**) represents the bio-inspired lamellar chitosan scaffold with an ordered porous structure with excellent mechanical properties, good cell-compatibility and promotion of vessel formation and gingival tissue regeneration in vivo. In addition, the LCS induces macrophage differentiation to M2 macrophages, which is thought to play an important role in tissue regeneration. Finally, (**D**) shows scaffolds produced from gelatin and alginate with microporous structures and interconnected microchannels.

Natural polymer-based scaffold materials should meet some requirements such as good biocompatibility, suitable mechanical properties, satisfied porosity, controlled degradability, and pore size ~5–200 μm. Human tissues span a broad spectrum of mechanical properties, where the stiffness of soft tissues typically ranges from 1 kPa (e.g., brain) to ~1 MPa (e.g., nerve and cartilage) [66]. Over the past decades, numerous approaches have been developed for fabricating natural polymer-based scaffolds, such as electrospinning, freeze-drying, and 3D printing. More details about such processes can be found in Chen et al. [70]. Figure 9 shows a schematic representation of natural-based scaffolds obtained by the mentioned methods.

Another important issue is that the transplant waiting list, which, in most cases takes from 3–5 years [71,72], leads to many deaths due to a delay in the waiting list for transplants. In this sense, tissue engineering and regenerative medicine provide biomedical engineers and doctors with appropriate strategies for replacing dysfunctional tissues/organs with biomaterials. There are a lot of different microstructures formed by the polymers (films, nanofibers, and hydrogels). The main objective is to eliminate surgical procedures, hence minimally invasive methods are preferred. In this sense, injectable hydrogels take a lot of attention [73] due to their unique characteristics. In this family, collagen, gelatin, elastin (polypeptide hydrogels (drug delivery, tissue engineering, and implants) and alginate, cellulose, glycosaminoglycan (polysaccharide hydrogels (drug delivery, tissue engineering, and biomed devices)) are used, as well as nucleic acids (drug delivery, tissue engineering, cancer therapeutics, and biosensing). Independently of the polymer selected, the polymer must exhibit biocompatibility and a controlled degradation rate.

**Figure 10:**
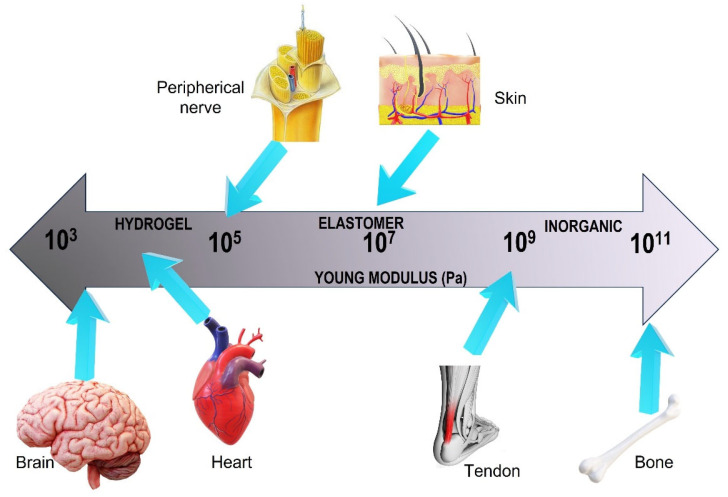
Young modulus scale and the respective structures. Inorganic materials are usually where the mechanical requirement is high while hydrogels and elastomers are employed where a softer material is needed. Adapted from [71].

There are a lot of biopolymers that can be used as tissue scaffolds. The biopolymer selected is dependent on the given tissue engineering purpose because some features must be reached such as proper microenvironment facilitating, cellular activity, growth, adhesion, differentiation, and proliferation. Zararintaj et al. [74] developed a review of biopolymer-based composites for tissue engineering applications such as neural tissue engineering, cardiac tissue engineering, skin tissue engineering, bone tissue engineering, and cartilage tissue engineering. Figure 10 represents the schematic representation of Young’s modulus and the respective polymers for use in the human body.

## 4. Prosthetic Limbs

**Figure 11:**
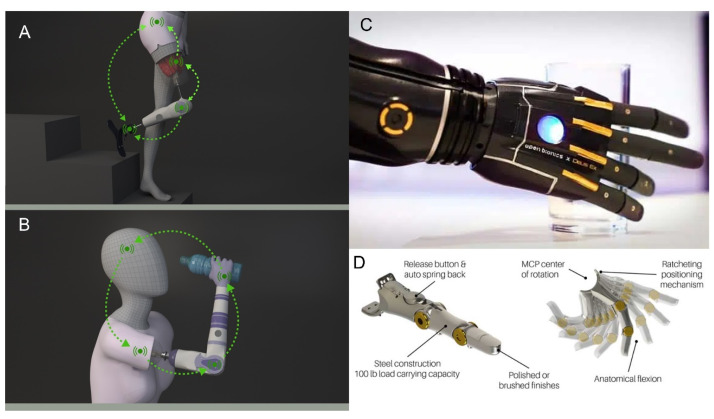
(**A**,**B**) shows the schematic representation of some bionic limbs used as bone-anchored prostheses used as implants directly used into the living bone for more stability [77]. (**C**) shows the current bionic hands (material did not inform) [78] and (**D**) shows bionic fingers using high-tech metallic appearance mounted on carbon fiber shell with a soft silicone inner socket [79].

With the advance of technology and science, a combination of efficiency with comfort is more easily obtained compared to earlier years. Naturally, science and engineering are in constant evolution, but future innovations in this area also depend on amputees’ demands and healthcare funding. There are some issues regarding the causes and impact of the amputated ones. Since amputation is a permanent disfigurement, some people do not face it well. Even when the amputation leads to a relief of pain, in all cases is required an adaptation time, which varies from person to person. Another point is that people from developed countries have more conditions to obtain prosthetic limbs compared to third-world countries. The great advance in technology was driven largely by the amputees’ demand [75] is the world-leading bionic hand and has the ultimate technology in prosthetic hands. Great progress in the bionic arms and hands, bionic legs and feet, and assistive robotic gloves among others can be found on [76,77]. Figure 11 shows the schematic representation of the products.

**Figure 12:**
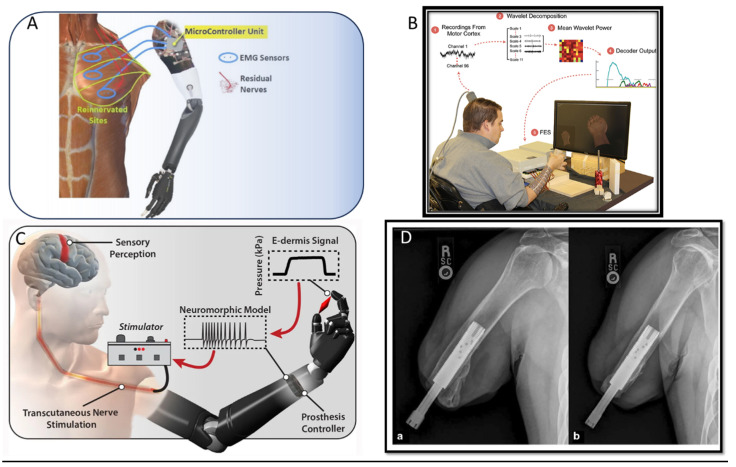
Some examples of advances in prosthetic limbs. (**A**) Artificial arm containing electromyogram (EMG) sensors that are inserted into reinnervated sites onto the human body [85], (**B**) Control of seven functional hand movements using cortically controlled transcutaneous muscle stimulation in a person with tetraplegia [86] (**C**) Prosthesis to perceive and transmit the feeling of pain [84,86], (**D**) Osseointegration [84].

Myoelectric prosthetics [80,81] are a branching of the upper prosthetic limbs that are based on the regulating nerves to move before amputation and have been reported to have increased dexterity, grip, and force [82]. Myoelectric technologies are available for all levels of upper limb loss, including myoelectric fingers, myoelectric hands, myoelectric elbows, and myoelectric hooks [83]. Many surgical innovations have improved the functionality of myoelectric prostheses: targeted muscle reinnervation (TMR) and regenerated peripheral nerve interface (RPNI) are two examples. More details can be found in Bates et al. [84] The restoring sensation is another concern about amputees. The ability to feel the same sensations is another hard task. This ability can be sensed visually, acoustically, or using some residual limb sensation. If tactile feedback is generated by activation of the harness when opening the terminal device or when flexing the elbow by a body-powered limb. Independently of the feedback the important is for the patient to feel self-identified with the prosthesis. Stimulation of the somatosensory cortex with intracortical microstimulation (ICMS), surface-level sensors integrated into prosthetic skin, and haptic are some methods used to improve the stimulation. Finally, osseointegration is an alternative to complications and restrictions inherent to socket-based prostheses, or a short residual limb. One drawback is the sweating that can alter the location of the electrodes and interfere with signal transmission, thus limiting the efficacy of the myoelectric device besides improving functionality, durability, and freedom of motion in the prosthesis as well as vibrotactile and pressure feedback secondary to osseoperception. Figure 12 shows a schematic representation of some simulation methods.

Regarding scientific papers, some studies can be found in the literature. Kumar et al. [87] studied natural fiber-reinforced polymers in knee prostheses due to their greater flexibility design and lighter weight that provide higher specific strength and stiffness compared to conventional biomaterials. Biddiss et al. [88] studied electroactive polymeric sensors in hand prostheses using ionic polymer metal composites to demonstrate the potential for prosthetic applications. Khare et al. [89] verified the influence of different resins on the physico-mechanical properties of hybrid fiber-reinforced polymer composites used in human prosthetics.

## 5. Future Trends

A great deal of progress was made regarding hard and soft tissues and prosthetic limbs using polymers and other conventional materials as BMs. With the advancement of technology and science, more and more products are being developed. An enormous variety of products have been successfully applied to different fields, leading to better mechanical performance allied with maximum comfort.

Gene therapy will likely become easier to use in the future, potentially making it more effective and labor-saving. To maximize patient benefit, it is most likely that a variety of bone-mending methods and treatments would be used together. To improve this field of study, more needs to be learned than just new methods for bone mending. The surgeon’s part in this procedure is equally crucial. To provide the patient with the best chance of a successful outcome, the surgeon must stay up to date on the latest procedures. He or she should first recognize a high-risk patient before adjusting the course of treatment to meet the needs of that patient [90].

The main BMs employed are ceramic or titanium alloys because they have the high mechanical performance required for dental applications. For dental implant devices, new titanium alloys (Ti-Zr and Ti-20Nb-10Zr-5Ta) are becoming more popular. Ceramic dental implants commonly fill the role of titanium implants due to their tooth-like color, mechanical properties, biocompatibility, and low plaque attraction. To advocate ceramic implants for routine clinical usage, there is, however, scant scientific proof [91]. This motivates researchers to hunt for novel materials, especially polymeric ones since they may have been used for medical applications. The most promising polymer is poly-ether-ether-ketone (PEEK), which is already utilized in orthopedics, traumatology, and calvarial reconstructions. For patients with low-stress tolerance and sensitivity to metallic materials, PEEK’s composites can be the perfect implant and restorative materials. PEEK has several advantages for dentistry. However, it is unclear whether the manufacturing process is best for PEEK usage in dentistry. For instance, 3D printing has the potential to produce mechanically superior restorations of architectural complexity, but more research is required to assess this manufacturing method’s accuracy. The processing techniques employed in 3D printing as well as postprocessing treatments can influence the crystallinity of PEEK materials. Insufficient mechanical properties may result from low crystallinity, while deformation may result from high crystallinity. It is important to look into how the material’s mechanical properties and deformation are impacted by crystallinity in order to create a balance of traits. extraordinary biocompatibility, significant osteoconductive and osteoinductive activity, extraordinary anti-inflammatory and anti-infective qualities, together with excellent mechanical properties, are all required for clinical implants to be successful. A new generation of PEEK materials with good mechanical qualities, osseointegration capabilities, and antibacterial properties needs to be further investigated by researchers. The majority of the studies that are now accessible on the bonding behavior of PEEK have only been conducted in vitro, and there are not any clinical trials using molded specimens. Future research should concentrate on how the oral environment affects bond strength and bond microleakage in order to evaluate the clinical viability and long-term performance of PEEK given the complexity of the oral environment. To ascertain the impact of various manufacturing processes and technical elements, such as printing temperature, speed, and layer thickness, on PEEK’s adhesive properties, additional clinical study is required. The clinical performance of PEEK materials is encouraging and positive overall. Future lengthy controlled clinical studies should be conducted to provide more fruitful outcomes for the usage of PEEK in dentistry [92].

Tissue engineering presents some prospects and future directions [93]. The gap between the number of patents (more than 9000) and publications (more than 10,000) with academic-industry collaborations, which increases the positive impact of scientific discoveries on patients in the clinic, is one of the major difficulties. The demise of the scaffolds’ cells after they are implanted in the body is a significant obstacle. This drawback can be minimized by developing a mechanism to deliver O_2_ into the engineering tissues by using O_2_-generating biomaterials. The enhancement of angiogenesis, accurate cell positioning in printed constructs and angiogenic growth factors can play important roles to avoid failure of engineered tissues or implanted constructs. To ensure continuous blood flow to specified constructs, methods for prevascularizing or vascularizing scaffolds using microsurgery have been investigated. The need for a functional vascular network increases with the complexity and size of the target tissue or organ. These circulatory networks could support the grafts in the period immediately following production.

Even though much of the early tissue engineering research was proof-of-concept and showed function, they typically used immune-deficient mice since healthy immune responses are useless in these animals. A challenging issue is the immune response to the design in immunocompetent animals after implantation. This response includes potential allogeneic cell reactions as well as non-specific inflammatory reactions to matrix constituents. There have been many approaches established to deal with these problems, including the use of autologous cells and the use of biocompatible materials. In addition, the exploration of autologous native extra-cellular matrix (ECM) derived materials has been pursued. It has been examined whether the use of immune response-modulating substances, such as anti-inflammatory compounds embedded or integrated with the biomaterial, will improve reactions to implanted biodegradable materials. A fascinating technique is to create cellular structures devoid of any unnecessary parts. Recent developments in the use of in situ tissue regeneration can be taken advantage of as an alternative to ex vivo engineering, helping to avoid many of the current problems with that method.

In situ tissue engineering, which can be performed by providing an in vivo environment that promotes the regeneration of a person’s own local cells, will probably receive greater attention in future studies. This can be conducted in a number of ways, such as by reprogramming cells using transcription factors and RNA-based methods or by interacting with immune cells, progenitors, or stem cells utilizing bioresponsive materials. In addition, the use of in situ and intravital 3D bioprinting would increase our ability to provide regenerative therapies in a less invasive or minimally invasive manner.

Future development of more individualized and tailored treatment modalities is made possible by the use of iPSCs (induced pluripotent stem cell) and stem cell-derived EVs (Extracellular vesicles), as well as unique implants made utilizing 3D printing. These new techniques for tissue and cellular engineering can help with the development of autologous endocrine tissues, such as those needed for the treatment of diabetes, as well as musculoskeletal tissues crucial for craniomaxillofacial reconstruction.

One area of tissue engineering that is progressing is the use of electroconductors. Future development of more individualized and tailored treatment modalities is made possible by the use of iPSCs (induced pluripotent stem cell) and stem cell-derived EVs (Extracellular vesicles), as well as unique implants made utilizing 3D printing. These new techniques for tissue and cellular engineering can help with the development of autologous endocrine tissues, such as those needed for the treatment of diabetes, as well as musculoskeletal tissues crucial for craniomaxillofacial reconstruction materials, which have numerous uses, including neurological tissue engineering. The integration of sensors, actuators, connectivity, and remote-control capabilities will improve patient care by enabling real-time monitoring of implanted constructions and prompt intervention by giving the right treatment at the right time. The Internet of Things advancements will help with diagnosis, treatment planning, implant installation, follow-up, and optimization.

Numerous investigations on examining tissues, creating disease models, and testing medications have been published as a result of improvements in microfluidic OoC (Organ-on-a-chip) systems. However, there are still plenty of unexplored potential uses for them that need to be investigated in order to further tissue engineering for regenerative medicine. Additionally, OoC systems will improve our capacity to conduct cell culture experiments in a dynamic 3D environment that can replicate the in vivo microenvironment. On microstructures made with 3D printers, cell growth and spread can be seen in a controlled manner, and real-time processes may be mimicked. Furthermore, we will be able to test and select the best cell type for use in the creation of particular tissues and specified therapeutic applications thanks to the usage of such sophisticated in vitro systems.

Future tissue engineering application studies will focus mostly on the use of smart BMs, stem cell research, the development of nanotechnology, novel biofabrication techniques, and the integration of synthetic biology breakthroughs. It will become simpler to mimic tissues and organs, especially as more stem cell research is conducted and published. More studies in this area may be able to address the problems with safety and effectiveness associated with some stem cell types, including iPSCs (induced pluripotent stem cells) and MSCs (Mesenchymal stem cells). The optimal methods to separate, and modify these cells ex vivo, and culture them need to be determined through additional research. The selection and design of BMs that are appropriate for the target tissue and organ presents one of the most significant challenges in tissue engineering applications. In addition, the harmony and integrity of the cells with BMs have made the use of biological materials as scaffolds useful for the integration of organs with 3D systems. Increasing the utilization of technical applications like computer modeling, artificial intelligence, OoC platforms, and 3D printing will be crucial in this situation for understanding how cells and tissues interact with biomaterials in vivo. As a result, tissue engineering applications using the new generation of biocompatible smart materials will be possible, and real-time patient needs will be met [93].

## 6. Limitations and Gaps

The use of changed tissue products may be related to worries about the safety of the cell, material, and molecular sources during the phases of retrieval, processing, storage, transit, and application. One of the most important ethical factors is the origin of the cells, such as xenogeneic grafts, chimera organisms, or ESCs (Electronic Stability Control System). Furthermore, issues relating to the risky, unproven, and untested stem cell therapies applied by unlicensed clinics need to be addressed. Moral concerns for applications and making the therapy available when needed and for those who need it are also essential, given the lack of sufficient organs and tissues needed to perform essential activities and stop the mortality of patients on the waiting list. Ethical concerns pertaining to clinical trials must be thoroughly investigated and addressed. It might be difficult to establish appropriate control groups for clinical trials due to ethical constraints. As a result, when applications are made to regulatory authorities for approval, outcomes should be examined correctly.

Health service providers and insurance companies might not offer or accept the processes due to budgetary considerations or the existence of other therapeutic alternatives. Because of financial limitations or the availability of other therapeutic options, health service providers and insurance companies may choose not to offer or authorize engineered tissue products. This is another barrier to the widespread use of engineered tissues. The level of clinical and patient acceptance will also have an impact on how modified tissue treatments are used in the future. Therefore, patient education, marketing, safety evidence, and efficacy evidence will all have an impact on wider clinical implementation. Products made from created tissues present another obstacle to the widespread use of synthetic tissues. Future adoption of modified tissue treatments will depend on how well-liked they are by medical professionals and patients. Therefore, patient education, marketing, safety evidence, and efficacy evidence will all have an impact on wider clinical implementation. Safety, efficacy, cost, and insurance company coverage for uses where alternatives are unavailable, ineffective, or more expensive are further contributing considerations. For specific clinical reasons, such as the treatment of facial lesions in younger people and extensive burn wounds, there is a market for engineered tissue products.

Many engineered tissues are small because of restrictions. This is partly because it is difficult to feed deeper constructs and bigger productions require vascularization. It remains challenging to create constructs in sizes that are clinically helpful, despite the most recent developments in 3D bioprinting for tissue engineering. Other strategies, such as printing supportive structures or printing into a supporting sacrificial material, have been developed to get around this. In the latter, it is believed that more substantial structures will result. To support tissues while they are healing, it is still necessary to obtain mechanically stable constructions that can maintain their physical and mechanical qualities for an extended period. Most challenges in this sense are related to constructs intended for use in hard tissue such as bone. Important strategies can be sought by combining acellular frames and scaffolds with cellular constructs, as has been suggested earlier.

## 7. Conclusions

The advantages of tissue engineering were successfully established in early short- to medium-term animal testing. The function is not always maintained, as later investigations using pancreatic endocrine and hepatic tissue engineering approaches showed. Therefore, strategies to increase the functionality and survival of implanted synthetic tissue constructs were investigated. The durability of the implanted modified tissue constructs needs to be demonstrated through long-term investigations. Recent advancements in in vivo imaging and cell tracking allow for better evaluation and monitoring of the post-implantation phase. Sensor technology is an emerging area that can be taken as an enabling tool to advance our capabilities in monitoring our implants further and pursue timely intervention if needed.

## Figures and Tables

**Table 2:** Characteristics of PEEK-derived materials proposed for use in oral implantology. Reproduced with permission from Ref. [61]. Copyright © 2015, Springer Science Business Media New York.

Implant Type	Study Type	Model Used	Results
*pPEEK* CFR-PPEK	In vivo	Dog femur	BIC: *pPEEK* < Ti; CFR-PEEK > Ti
*pPEEK* CFR-PEEK	In vivo	Dog mandible	BIC: *pPEEK* < Ti; CFR-PEEK < Ti
CFR-PEEK	In silico	FEA	Stress peaks: CFR-PEEK > Ti
CFR-PEEK GFR-PEEK	In vivo	ISO 14,801 protocol	Stress shielding effects: CFR-PEEK < Ti rods; GFR-PEEK < Ti rods
*HAcCFR-PEEK* CFR-PEEK	In vivo	Rabbit femur	Interfacial shear strength: *HAcCFR-PEEK* = grit blasted Ti allow with *HA; HAcCFR*-PEEK > CFR-PEEK
*pPEEK*	In vivo	MG-63 cells	Proliferation rate: *pPEEK* < Ti; mRNA processing: *pPEEK* < Ti
nTiO_2_-PEEK	In vitro and in vivo	MG-63 cells and beagle dog tibia	Bioactivity: nTiO_2_/PEEK > Ti
*St-HAcCFR-PEEK*	In vitro	MG-63 cells	Bioactivity: *St-HAcCFR-PEEK* > Ti
*nHAcPEEK*	In vivo	Rabbit femur	Osseointegration: *nHAcPEEK* > Ti Implant loss: *nHAcPEEK* > Ti
*eTicPEEK*	In vitro and in vivo	MC3T3-E1 cells and rabbit tibia	Cell proliferation: *eTicPEEK* > Ti BIC: *eTicPEEK* > Ti

PEEK poly-ether-ether-ketone, pPEEK pure PEEK, BIC bone-implant contact, Ti titanium, CFR-PEEK carbon-fiber-reinforced PEEK, GFR-PEEK glass-fiber-reinforced PEEK, FEA finite element analysis, HAcCFR-PEEK hydroxyapatite (HA)-coated CFR-PEEK, St- HAcCFR-PEEK strontium reinforced HA-coated CFR-PEEK, nano-TiO_2_/PEEK, PEEK combined with nanoparticles of Titanium dioxide, nHAcPEEK nanocrystalline HA-coated PEEK, eTicPEEK electron beam pure Titanium-coated PEEK.

## Data Availability

Not available.
